# Cervicovaginal Müllerian papilloma malignant transformation in a prepubertal girl

**DOI:** 10.1136/jclinpath-2018-205612

**Published:** 2019-07-25

**Authors:** Juan Zou, Lingping Xie, Xue Xiao, Lian Xu, Fan Yang, Tong Qiu, Kaixuan Yang

**Affiliations:** 1 Department of Pathology, West China Second University Hospital, Sichuan University, Chengdu, China; 2 Key Laboratory of Birth Defects and Related Diseases of Women and Children (Sichuan University), Ministry of Education, West China Second Hospital, Sichuan University, Chengdu, China; 3 Department of Pathology, Cheng Du Shang Jin Nan Fu Hospital, West China Hospital, Sichuan University, Chengdu, China; 4 Department of Gynecology and Obstetrics, West China Second University Hospital, Sichuan University, Chengdu, China; 5 Laboratory of Stem Cell and Tissue Engineering, West China Second University Hospital, Sichuan University, Chengdu, China

**Keywords:** gynaecological pathology, cervical cancer, oncology

## Clinical question

A 13-year-old girl presented with irregular vaginal bleeding for 2 years and aggravated 10 days. Anal examination showed a hard vaginal mass. On the MRI, a 7.1 cm×6.3 cm×5.5 cm mass at the upper part of the vagina was seen, the relationship with cervix was unclear. The biopsy revealed a malignant tumour with massive necrosis, tended to be rhabdomyosarcoma or the other sarcoma. Because the clinical stage was International Federation of Gynecology and Obstetrics IIA, neoadjuvant chemotherapy took before the radical operation.

Review the high-quality, interactive digital Aperio slides at http://virtualacp.com/JCPCases/jclinpath-2018-205612_1/ and http://virtualacp.com/JCPCases/jclinpath-2018-205612_2/ and consider your diagnosis.

## What is your diagnosis?

- Mesonephric adenocarcinoma
- Serous carcinoma
- Malignant mixed Müllerian tumours
- Müllerian papilloma malignant transformation to endometrioid carcinoma
- Benign Müllerian papilloma

­

The correct answer is after the discussion.

## Discussion

Müllerian papilloma of the genital tract is rare. It mainly occurs in prepubertal girls ranging from 1 to 9 years old, and the major symptom is non-cyclical vaginal bleeding or discharge. Since James^1^ reported the first case in 1951, only 56 cases of Müllerian papilloma were reported,^2^ all cases are benign and have an excellent prognosis after the local excision, unless one with clear cell carcinoma transformation after 10 recurrences when she was 50 years old.^3^

**Figure 1:**
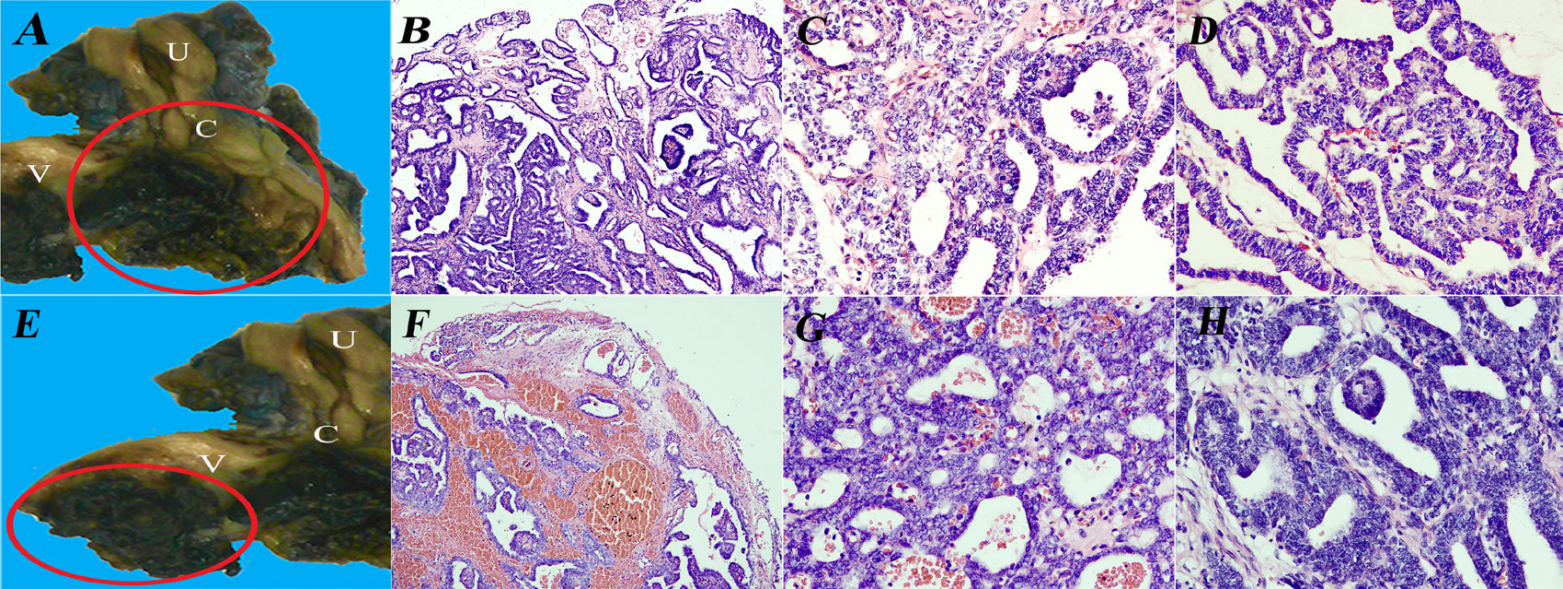
The macroscopy and histopathology of the cervical mass. (A) Macroscopy of the uterine and vagina, the cervical mass was marked by the red circle. (B) Scanning view of the cervical tumour, the benign and malignant region included, ×100. (C, D) High-power magnification showing the malignant region with branching and sieve architecture, ×400. (E–H) The macroscopy and histopathology of the vaginal mass. (E) The vaginal mass was marked by the red circle. (F) Scanning view of the vaginal tumour, the benign and malignant region included, ×100. (G, H) High-power magnification showing the malignant region with sieve and branching architecture, ×400. C, cervix; U, uterine corpus; V, vagina.

**Figure 2:**
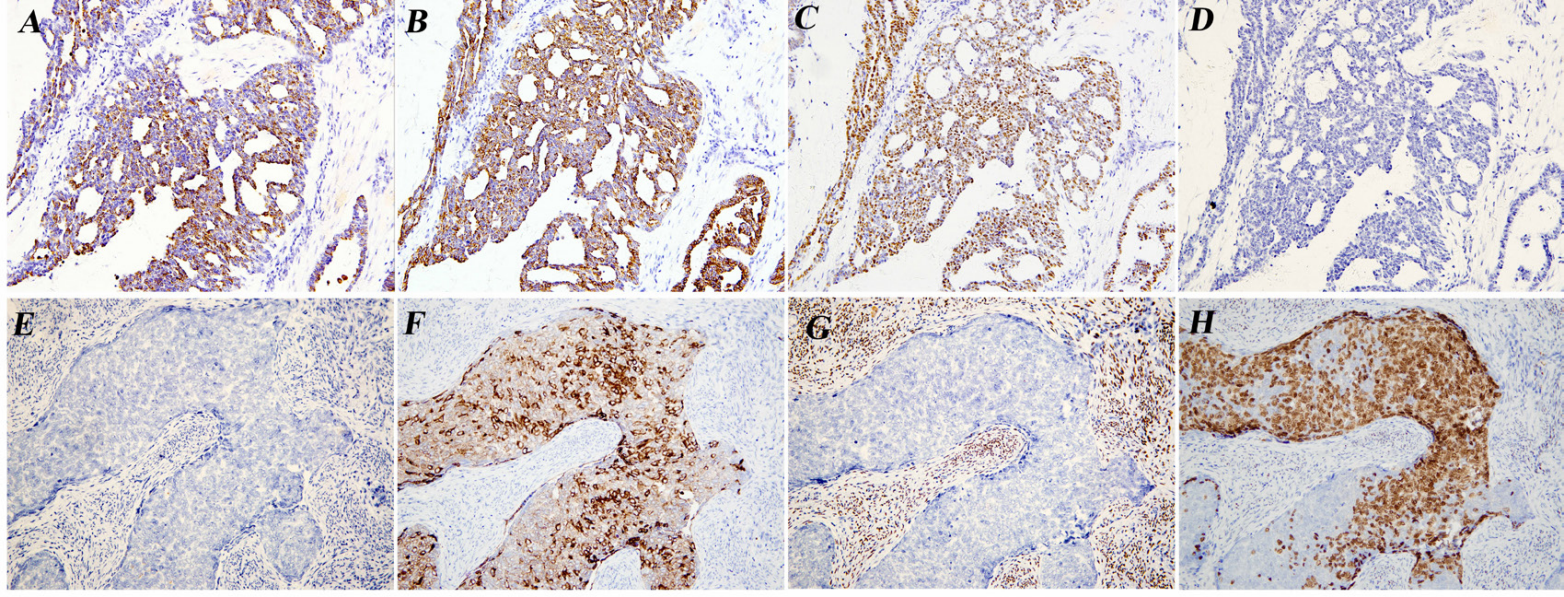
Tumour cells were positive for CK7 (A, E), epithelial membrane antigen (EMA; B, F), oestrogen receptor (ER; C, G) and progesterone receptor (PR; D, H) ×200.

This is the first case of Müllerian papilloma malignant transformation in a prepubertal girl, the endometrioid carcinoma differentiation(figure 1C,D,G,H) has not been reported. The existence of benign Müllerian papilloma, borderline and malignant transformation may be the main diagnostic standard.(figure 1B,F) The exogenous papillary architecture, the expression of CK7, CA125, oestrogen receptor, progesterone receptor and negative for calretinin, vim, CD10^1 4 5^ can be differentiated from mesonephric adenocarcinoma.(figure 2) On biopsy, the solid malignant region may cause misdiagnosis of malignant embryonal rhabdomyosarcoma which was a more common and aggressive tumour in this age group. Mostly, the cells are positive with epithelial membrane antigen and negative for desmin, myogenin and myoD1.^2^

The cervix and the vagina were coinvolved in our case. Both masses had the benign to malignant region and were isolated from each other with a distinct boundary.(figure 1A,E) We could not exclude the possibility of the double primary.

The Müllerian papilloma malignant transformation had not been reported in the prepubertal girl, there may be some relationship with the development abnormity as this case was combined with uterine hypoplasia.

## Answer

D. Müllerian papilloma malignant transformation to endometrioid carcinoma.

**Take home messages**

- The Müllerian papilloma malignant transformation is rare; the existence of benign Müllerian papilloma, borderline and malignant transformation may be the main diagnostic standard.
- The Müllerian papilloma malignant transformation can perform all kinds of adenocarcinoma of the female genital tract as the Müllerian papilloma gland epithelium is primitive.
- Useful immunohistochemical markers are helpful to diagnose and determine the differential diagnoses.
- The Müllerian papilloma malignant transformation is available in both the old and prepubertal woman, we should take attention to the vaginal bleeding of prepubertal girl for early detection and treatment.
